# The Biopsychosocial Model and Perinatal Health Care: Determinants of Perinatal Care in a Community Sample

**DOI:** 10.3389/fpsyt.2021.746803

**Published:** 2021-11-17

**Authors:** Zoe T. Duberstein, Jessica Brunner, Lisa S. Panisch, Sanjukta Bandyopadhyay, Carrie Irvine, Jenna A. Macri, Eva Pressman, Loralei L. Thornburg, Ellen Poleshuck, Keisha Bell, Meghan Best, Emily Barrett, Richard K. Miller, Thomas G. O'Connor

**Affiliations:** ^1^Department of Psychology, University of Rochester, Rochester, NY, United States; ^2^Department of Obstetrics and Gynecology, University of Rochester, Rochester, NY, United States; ^3^School of Social Work, Wayne State University, Detroit, MI, United States; ^4^Clinical and Translational Science Institute, University of Rochester, Rochester, NY, United States; ^5^Department of Psychiatry, University of Rochester, Rochester, NY, United States; ^6^School of Public Health, Rutgers University, Piscataway, NJ, United States; ^7^Department of Neuroscience, University of Rochester, Rochester, NY, United States; ^8^The Wynne Center for Family Research, University of Rochester, Rochester, NY, United States

**Keywords:** prenatal care utilization, postpartum, attendance, biopsychosocial, maternal-fetal health

## Abstract

Insufficient care in the perinatal period is associated with poorer maternal health, poorer perinatal outcomes, infant mortality, and health inequalities. Identifying the sources of and reducing the rates of insufficient care is therefore a major clinical and public health objective. We propose a specific application of the biopsychosocial model that conceptualizes prenatal and postpartum care quality as health markers that are influenced by psychological factors and family and social context. Clinic attendance data were abstracted from the electronic medical records of *N* = 291 participants enrolled in a longitudinal pregnancy cohort study of healthy women who have been followed since the first trimester; the Kotelchuck Index (KI) was calculated as an index of perinatal care utilization. Detailed prenatal psychological, social, and sociodemographic data were collected from self-report questionnaire and interview. Bivariate analyses indicated socio-demographic (e.g., race), psychological (e.g., response to perceived racism, affective symptoms, trauma experience), and social and family context (e.g., social support, family size) significantly influenced pre- and post-natal care utilization. Multivariate logistic regression analyses, adjusting for medical complications, identified social and family context as robust predictors of perinatal care utilization. The findings underscore the need for biopsychosocial models of health care and highlight several potential strategies for improving health care utilization.

## Introduction

The biopsychosocial model, most closely associated with George Engel (1), was proposed as an alternative to the then-dominant biomedical model for understanding health and delivering healthcare. What distinguished the biopsychosocial model was an emphasis on individual needs, the social context of health and heath care, and most especially the dependencies between an individual's biological processes underlying health and the social and cultural systems in which she/he is embedded. This model spurred a major conceptual shift: its foundation underlies current research paradigms (e.g., “social determinants of health”) and values for health care delivery (e.g., “patient-centered” care). Nonetheless, the model has been criticized for being difficult to operationalize (2).

Opportunities provided by a biopsychosocial model to improve health and health care delivery may be especially important in the perinatal period. That is because of the high prevalence and burden of pregnancy-related health conditions such as preeclampsia and gestational diabetes. More broadly, the rates of pregnancy-related morbidity and mortality are increasing in the United States (3) and the World Health Organization has stated that “ending preventable maternal mortality remains an unfinished agenda and one of the world's most critical challenges” (4). Applications of the biopsychosocial model's focus on psychosocial and cultural contexts may provide clues to improving perinatal health care and particularly health inequalities, which are pronounced and persisting for maternal morbidity and mortality. The application of a biopsychosocial model to perinatal health may also benefit both maternal and child health given two decades of reliable evidence from large-scale studies linking prenatal maternal psychological well-being with child's behavioral and physical health (5). We propose an application of the biopsychosocial model to perinatal health care that conceptualizes prenatal clinic attendance and utilization as a health marker that is influenced by psychological, psychosocial, and sociodemographic factors.

The Kotelchuck Index (KI) or the Adequacy of Prenatal Care Utilization (APNCU) is a validated and widely used measure of adequacy of prenatal care; it is identified as the recommended measure in the PhenX Toolkit. The KI classifies prenatal care utilization into inadequate (<50%), intermediate (50–79%), adequate (80–109%), and “adequate plus” (also referred to as intensive or excessive care, at 110% or greater), based on the percent of American College of Obstetricians and Gynecologists (ACOG) recommended visits received. Inadequate (usually combined with intermediate) care during pregnancy is associated with poorer maternal health (e.g., hospitalizations after delivery), lower birth weight, higher rates of prematurity, and infant mortality (6, 7). There remains some controversy about any causal connection between prenatal care utilization and maternal and child health outcomes. Nonetheless, improving quality and access to prenatal care remains a high clinical and public health priority and a widely suggested means for reducing disparities in maternal and child health outcomes. It is therefore an important target for clinical research.

Several risk factors of inadequate prenatal care utilization have been identified; much of the focus and most of the consistent findings concern socio-demographic characteristics. Women with low socioeconomic status, defined according to Medicaid eligibility, education, and neighborhood deprivation are more likely to receive inadequate prenatal care (8). Care utilization is also frequently associated with race and ethnicity. Black and Latinx women are at a greater risk for late initiation of care, not obtaining any care, and receiving inadequate prenatal care (8–10). In fact, maternal morbidity and mortality is one of the most acute examples of health disparities in the US. The reasons for associations between perinatal care utilization and socio-demographic or socio-economic factors are not well-understood and are likely to be complex and confounded. More fundamentally, sociodemographic factors are not proximal causes of variation in perinatal care utilization but may point to underlying causes, such as systemic racism. For example, Black and Latinx women experience several types of economic and social barriers that may explain lower rates of adequate prenatal care (11). There is also evidence that racism and perceived discrimination may predict lower rates of adequate prenatal care in Black and Latinx women (8, 12).

Socio-demographic and socio-economic factors are confounded by psychological and social context, e.g., insofar as stress associated with economic deprivation and discrimination can contribute to and be exacerbated by adverse mental health conditions (13, 14). Accordingly, by focusing on the inter-related connections between social, demographic, and psychological factors, a biopsychosocial model may provide a plausible, more complete, and more practical explanation for variation in perinatal care utilization. Moreover, a biopsychosocial model may provide plausible intervention targets to improve perinatal care utilization, i.e., to the extent that identified psychological or social factors are more modifiable than sociodemographic characteristics. Our development of a testable biopsychosocial model focused on identifying hypothesized sources of risk for poor prenatal care utilization from the extant literature—which typically examines single or limited factors in isolation—and considering possible confounding among these sources of risk.

There is a limited but growing evidence base linking KI with social and cultural context and psychological processes hypothesized by a biopsychosocial model (15, 16). Psychological symptoms, including impairments significant enough to warrant diagnosis, may directly or indirectly alter prenatal care utilization. For example, women with a psychiatric diagnosis, including substance use disorders, may enter prenatal care late and/or receive inadequate care (15, 16). The nature of the effect is not consistent across all studies, however, with some (17) finding that affective symptoms are associated with less than adequate care, but others (18) reporting affective symptoms increase care; other reports are inconclusive (19, 20). Variation in effects reported may be explained by the severity of and type of symptoms and confounding health risk and social and cultural context.

Interpersonal and relationship context are significant components of a biopsychosocial model that may also shape perinatal care utilization. One of the most active areas of research concerns interpersonal violence (IPV). Wolf et al. (21) and Cha and Masho (22) reported that preconception and/or prenatal IPV was associated with inadequate prenatal care. One meta-analysis (23) found that women who experienced IPV had a decreased likelihood of attending at least four prenatal care visits during pregnancy; a separate meta-analysis (24) indicated that women with past-year experiences of IPV had a high likelihood of delaying or never seeking prenatal care. However, meta-analyses expose the variation in effects across study, perhaps related to the nature of how violence and abuse is assessed; several studies did not find reliable associations between IPV and prenatal care utilization (25).

Although most of the concern in the scientific and clinical literature is on inadequate care, there is a small but growing literature on the demographic, social, and psychological factors associated with intensive or excessive prenatal care. Whether or not intensive prenatal care may reflect the same kinds of social and psychological processes associated with excessive care utilization outside of pregnancy is unclear. What is emerging is that excessive or intensive care is not super-adequate, but may instead signal different kinds of risk, and may also be non-optimal in terms of maternal and child health outcomes. For example, intensive or excessive care is associated with higher risk of preterm birth and low birth weight (10) as well as low readiness for parenting and high psychosocial risk (19, 26). It is possible these associations exist primarily because high medical risk pregnancies require more frequent visits and therefore fall into the intensive category of the KI. At a minimum, these findings suggest that research on prenatal care utilization needs to consider both inadequate and intensive care patterns, and that the associations between psychosocial and interpersonal factors and care utilization may not be monotonic.

A further feature of the study is consideration of both prenatal and postnatal care utilization. ACOG recommends a postnatal appointment by 12 weeks postpartum because it has obvious benefits for tracking early infant and maternal health, including postpartum depression (27). Few studies have examined the predictors of postpartum visit completion, and only one to our knowledge has factored in prenatal attendance data. That study (28) found that low income, enrollment in Medicaid, unemployment, multiple children, and missed prenatal visits were significantly associated with an incomplete postpartum visit. We aim to replicate and extend these findings.

In the current study we consider how a biopsychosocial model may be applied to perinatal care and consider how variation in care is affected by an individual's psychological, interpersonal, and social context. We capitalized on an ongoing, prospective longitudinal pregnancy cohort that has several design features for advancing research on perinatal care utilization. First, we sampled women from community and university prenatal clinics and enrolled participants at the first trimester; that meant that we are examining variation in prenatal care unconfounded with delayed prenatal care, a limitation in prior studies [but see (29)]. Second, the sample was medically healthy (i.e., not greater than normal risk at enrollment), which provides leverage for assessing psychosocial factors unconfounded with medical risk status. Third, the diverse sample has been assessed at each trimester with an extensive battery of psychological, health behavior, socio-demographic, and psychosocial measures. For example, in addition to assessing depressive and anxiety symptoms, we also measure pregnancy-related anxiety, which has been suggested to have distinct features and correlates with perinatal and child outcomes (30). We are therefore able to provide a detailed portrayal of the social and psychological contexts for prenatal care utilization, and analysis of competing explanations for variation in prenatal care utilization.

## Materials and Methods

### Sample

The study sample (Understanding Pregnancy Signals and Infant Development, “UPSIDE”) is a prospective longitudinal pregnancy cohort conducted at the University of Rochester Medical Center (URMC); it is part of the NIH Environmental influences on Child Health Outcomes (ECHO) program. Between December 2015 and April 2019, women were recruited in their first trimester of pregnancy from outpatient obstetric clinics affiliated with the University of Rochester. Eligibility criteria were: <14 weeks gestation; age 18 or older; singleton pregnancy; no known substance abuse or a history of psychotic illness; ability to communicate in English; not greater than normal medical risk and without major endocrine, metabolic or immune disorders. Women received prenatal care through URMC or a URMC affiliated clinic, making their clinic attendance records accessible to research staff. Participants were compensated for each research visit and provided transportation or compensated for parking if needed. The study is approved by the URMC Institutional Review Board; written consent was obtained from all participants. For the current study, we excluded women with incomplete clinic attendance data from the medical record and those who discontinued care within a URMC clinic during pregnancy; we also excluded women who developed major endocrine, metabolic or immune disorders after enrollment and women who did not have a live birth.

### Measures

Clinic attendance data were abstracted from the electronic medical record; demographic, psychological, social, and health data were collected from in-person interview or questionnaire at prenatal visits scheduled to coincide with a routine prenatal visit in Trimester 1, 2, and 3; data on child birth weight and gestational age were abstracted from the medical record.

#### Clinic Attendance

The number of visits scheduled and the outcome of each of these visits (completed, canceled/rescheduled, no-show) was recorded from the medical record. The Kotelchuck Index (KI) or the Adequacy of Prenatal Care Utilization Index (6) was calculated in the standard manner as the ratio of the observed number of completed routine prenatal visits to the expected number of visits. The expected number of prenatal visits is based on the guidelines published by the American College of Obstetrics and Gynecology (ACOG) and is adjusted based on total gestational weeks and timing of initiation of care. The ratio of observed to expected visits is categorized into one of five groups: no prenatal care, inadequate (<50%), intermediate (50–79%), adequate (80–109%), or intensive (110% or greater) care. For example, a woman who enters prenatal care in her first trimester and has a 40-week pregnancy is expected to receive 14 visits. None of the study participants received no prenatal care.

Postpartum visit attendance was analyzed as a binary variable, according to whether or not a postpartum visit was completed by 12 weeks postpartum, as recommended by ACOG. Women were considered to have not had a postpartum visit only if it was confirmed in the EMR.

Data was also collected on prenatal no-show visits. A visit was considered a no-show when a participant failed to attend her scheduled prenatal visit and did not call to cancel her appointment. More information on the methods and results for no-show data can be found in Supplementary Table 1.

#### Birth Outcomes

Birth weight (g) and gestational age (weeks) based on ultrasound or last menstrual period were abstracted from the medical record.

#### Health Predictors

Participant age, parity, pre-pregnancy body mass index (BMI), and smoking status (including vaping) was collected from questionnaire and interview at enrollment. A variable for pregnancy complications was created to encompass women who were diagnosed with pre-eclampsia, gestational diabetes, or gestational hypertension in the EMR.

#### Social Predictors

##### Demographics

Employment status (employed vs. unemployed; number of hours worked per week), education level (highest level attained), marital status (single vs. married/cohabiting), and number of household members (not including the participant) was collected from questionnaire and interview at enrollment. Self-reported race and ethnicity was recorded; we identified three groups sufficiently large enough for comparison: non-Hispanic White, non-Hispanic Black, Hispanic/Latinx; the small number of other groups and mixed race/ethnic groups were included in an “other” category for analysis purposes. The income to needs variable was calculated by taking a ratio of poverty level as determined by the US Department of Health and Human services (31) by annual income. Participants also indicated whether they utilized services from Women, Infants and Children (WIC), Medicaid, or other public assistance. Women were recruited from one of three types of obstetric clinics: clinics serving a high psychosocial need population (hereafter “Community Clinic”), general university obstetrics clinic, and a Midwifery Practice.

#### Psychological Predictors

##### Social Support

The Interpersonal Support Evaluation List (ISEL) (32) is a widely-used index of social support in the perinatal period that assesses perceived availability of instrumental and expressive support. Thirty out of the original set of 40 items were answered on a scale of 1 (definitely true) to 4 (definitely false), with higher scores indicating higher perceived availability of support. The ISEL was administered at the second trimester study visit.

##### Anxiety

The Penn State Worry Questionnaire (PSWQ) (33) is a widely-used 16-item instrument used to assess symptoms of anxiety and worry. Items range from 1 (not at all typical of me) to 5 (very typical of me); higher scores indicate more anxiety and worry.

##### Pregnancy-Related Anxiety

Pregnancy-related anxiety was measured using a modified version of the Pregnancy Related Anxieties Questionnaire-Revised (PRAQ) (30) Three items assess anxiety and worry about the pain of delivery; four items assess anxiety and worry about the baby's health.

##### Depression

The Edinburgh Postnatal Depression Scale (EPDS) (34) is a widely-used 10-item scale to assess depression in the perinatal period independent of physical symptoms that could be confounded by pregnancy. Items are scored 0–3; higher scores indicate more depression.

##### Interpersonal Violence

Interpersonal abuse and violence were assessed using a screener based on previously used tools (35, 36). The questions assess for current and past physical, sexual, and emotional abuse. The composite score (“interpersonal violence general”) was calculated by summing the three screening items. The IPV screening items were administered at the third trimester study visit.

##### Stressful Life Events

The Stressful Life Events scale (SLE) (37) is a 26-item scale that asks about stressful events that may have happened to the participant during the last year and was developed specifically for pregnant samples. We assessed the total number of events as the measure of stress. The SLE was administered at the third trimester study visit.

##### Discrimination

Three sections of the Experiences of Discrimination scales (EOD) (38) were adapted to measure discrimination based on race and ethnicity only. Response to Unfair Treatment is a 2-item measure assessing level of passivity in response to being treated unfairly based on race or ethnicity; higher score indicates greater passivity of response. The Discrimination scale asks participants to answer “yes” or “no” to whether they have experienced discrimination in 9 separate contexts; higher score indicates higher self-reported discrimination. The Everyday Discrimination scale contains 9 items to assess the frequency with which participants experience discrimination; higher score indicates less frequent discrimination.

### Statistical Analyses

The PSWQ, EPDS, and PRAQ were administered at each trimester; the final composites were averages of each of the three summed scores. After reporting descriptive statistics, we present bivariate analyses between the KI and obstetrics outcome and predictor variables. Significant predictors of prenatal care (*p* < 0.05) in bivariate analyses were included in a multivariate analysis to examine independent effects. We followed previous studies in categorizing prenatal care utilization as Inadequate/Intermediate, Adequate, and Intensive. For bivariate and multivariate results, Adequate care is the reference category. Two contrasts were examined: Adequate contrasted with Inadequate/Intermediate and Adequate contrasted with Intensive. For the multivariate analyses, data were analyzed using multinomial regression with Adequate as the reference category; odds ratio and significance is reported for each contrast. A similar strategy was employed for postpartum care utilization, with bivariate analyses preceding multivariate analyses. The dichotomized response for postpartum visits means that the sole contrast was between attendance at a postpartum visit (or not); For bivariate analyses using Analysis of Variance or chi-square analyses and multivariate analyses using logistic regression. All statistical analyses were performed in IBM SPSS Statistics (39). In addition to reporting analyses of the KI, we also report, for comparison purposes, parallel analyses for no-show visits (see Supplementary Table 1).

## Results

Our analytic sample was composed of 291 (of the 326) participants in the cohort who met the inclusion and exclusion criteria. The majority, 70%, of the women received Adequate care compared with Inadequate (*N* = 6, 2%), Intermediate (*N* = 57, 20%), and Intensive (*N* = 23, 8%). Descriptive statistics (Table 1) indicate that 1/3 of the participants were first-time mothers, and just over half of the participants identified as non-Hispanic White (54.6%). The psychosocial risk-enriched nature of the sample is indicated by the percentage with high school education or less (36%), Medicaid recipient status (44%), and single-parent status (42%).

**Table 1:** Sample descriptive data (*n* = 291^a^^,^^b^).

**Maternal characteristics^**c**^**	**Mean (SD)**	**Mix–Max**	***N* (%)**
Age (years)	28.7 (4.7)	18–41	
Pre-pregnancy BMI (kg/m^2^)	28.3 (7.1)	15.3–49.1	
Household size (persons)	3.3 (1.4)	1–11	
Ethnicity/race			
Non-Hispanic White			159 (54.6)
Non-Hispanic Black			75 (25.8)
Hispanic			33 (11.3)
Asian			10 (3.4)
Other^d^			14 (4.8)
Education			
< High school			9 (3.4)
High school			88 (32.8)
Some college			41 (15.3)
College degree			65 (24.3)
Post-college degree			65 (24.3)
Employment status			
Employed			208 (73.5)
Unemployed			75 (26.5)
Marital status			
Married/living as married			163 (57.6)
Single			120 (42.4)
Medicaid enrollment			
Yes			111 (43.5)
No			144 (56.5)
Nulliparous			
Yes			96 (33.0)
No			195 (67.0)
Smoking during pregnancy (any)			
Yes			89 (31.4)
No			194 (68.6)
Pregnancy complications			
Yes			31 (10.7)
No			260 (89.3)
Enrollment clinic			
General OB			60 (20.6)
Midwifery			87 (29.9)
Community clinic			144 (49.5)
**Infant characteristics** ^**e**^	**Mean (SD)**	**Min–Max**	
Gestational age (weeks)	39.4 (1.5)	32.1.1–42.7	
Birth weight (g)	3,362.6 (534.8)	1,280–4,730	

a *N = 326 Upside participants considered for inclusion. Participants were excluded if: they transferred prenatal care out of URMC during pregnancy, they did not have a live birth, they did not receive prenatal care within URMC affiliated system, they became screen failures after enrollment*.

b *N's for individual variables may differ slightly due to missing data*.

c *At time of enrollment*.

d *“Other” including more than one race and American Indian/Alaska Native and individuals self-reporting as “other”*.

e *See Figure 1 for graph of infant characteristics by KI category*.

Prenatal care utilization was significantly associated with birth weight and gestational age (Figure 1) in this healthy, normal risk sample. Bivariate analyses indicated that prenatal care utilization was associated with multiple socio-demographic, psychological, and family and social context variables; differences were observed for both Inadequate/Intermediate and Intensive care compared with Adequate care (Table 2). Inadequate/Intermediate care was associated with race/ethnicity: compared to non-Hispanic whites, an increased likelihood of Inadequate/Intermediate care was observed for non-Hispanic Blacks (*OR* = 2.01, 95% *CI* [1.05, 3.87], *p* = 0.036) (an increased likelihood of Inadequate/Intermediate care was observed for those in mixed or “other” group, *OR* = 3.10, 95% *CI* [1.20, 8.00], *p* = 0.019, but the interpretation is unclear because of the small size and heterogeneous composition). In addition, younger age was significantly associated with an increased likelihood of less than adequate care, as was larger household size (Table 2). Individuals who experienced Inadequate/Intermediate also differed from those who received Adequate care in reporting less social support (*OR* = 0.98, 95% *CI* [0.96, 1.00], *p* = 0.026) and less pregnancy-related anxiety (*OR* = 0.96, 95% *CI* [0.93, 1.00], *p* = 0.014). A more novel finding concerned discrimination: those women who reported a more passive response to unfair treatment were more likely to experience less than adequate care (*OR* = 1.56, 95% *CI* [1.01, 2.42], *p* = 0.044). However, discrimination based on the Everyday Discrimination scale was not associated with care utilization; in fact, only *n* = 4 individuals reported experiencing discrimination on a health care setting. Rates of Inadequate/Intermediate care varied across clinic site: attending the midwifery clinic significantly predicted a lower likelihood of less than adequate care in comparison to the community clinic (*OR* = 0.46, 95% *CI* [0.23, 0.94], *p* = 0.033).

**Figure 1:**
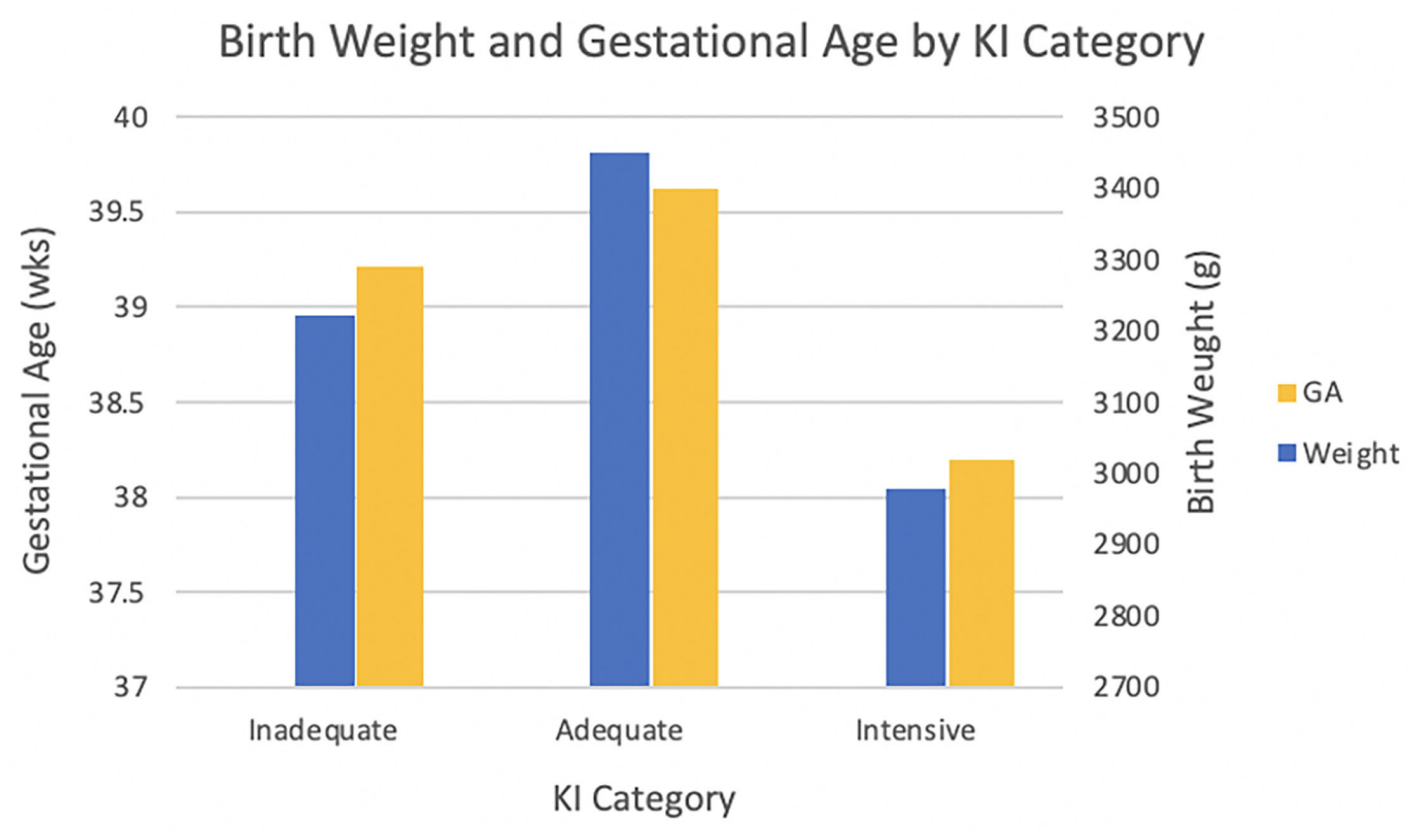
Differences in birth weight and gestational age based on level prenatal care. Adequate care babies (*M* = 3448.78 ± 504.19 g) had higher mean birthweight than Inadequate/Intermediate (*M* = 3222.49 ± 467.96 g) and Intensive care (*M* = 2977.57 ± 718.11 g) babies [*F*_(2, 288)_ = 11.57, *p* < 0.001]. The mean gestational age for Intensive care babies (*M* = 38.19 ± 2.84 wks) was earlier than that for Inadequate/Intermediate (*M* = 39.21 ± 1.17 wks) and Adequate care (*M* = 39.62 ± 1.36 wks) babies [*F*_(2, 288)_ = 10.31, *p* < 0.001].

**Table 2:** Bivariate logistic regression analysis predicting inadequate and intensive prenatal care vs. adequate prenatal care.

**Predictors**	**Less than adequate**	**Adequate**	**Intensive**		**Less than adequate vs. adequate care**	**Intensive vs. adequate care**
	**frequency (%)**			***N***	**OR (95% CI)**	**OR (95% CI)**
Age (years)	27.5 (4.86)	29.2 (4.48)	28.4 (5.1)	291	0.92^**^ **(0.87–0.98)**	0.96 (0.88–1.06)
Pre-pregnancy BMI (kg/m^2^)	28.6 (6.85)	27.9 (7.21)	30.26 (6.54)	291	1.01 (0.97–1.05)	1.04 (0.99–1.10)
Poverty income ratio	2.8 (2.13)	3.9 (4.40)	3.4 (2.85)	241	0.87 (0.75–1.00)	0.97 (0.84–1.11)
Household size (persons)	2.8 (2.05)	2.2 (1.21)	1.7 (0.86)	284	1.29^**^ **(1.08–1.55)**	0.70 (0.45–1.11)
Employed hours/week	36.0 (16.14)	34.8 (11.21)	39.8 (11.68)	204	1.00 (0.98–1.04)	1.03 (0.99–1.07)
Depressive symptoms	6.1 (5.00)	5.7 (4.45)	7.3 (3.84)	287	1.02 (0.96–1.09)	1.07 (0.98–1.17)
Pregnancy anxiety (regarding baby)	17.6 (9.48)	21.5 (11.04)	22.5 (11.03)	290	0.96^*^ **(0.93–0.99)**	1.01 (0.97–1.05)
Pregnancy anxiety (regarding labor)	17.6 (8.91)	19.6 (9.70)	21.3 (11.08)	290	0.98 (0.95–1.01)	1.02 (0.97–1.06)
Social support	95.0 (19.27)	100.9 (15.85)	103.1 (13.05)	263	0.98^*^ **(0.96–1.0)**	1.01 (0.98–1.05)
Worry symptoms	42.9 (12.67)	44.4 (13.19)	47.4 (9.64)	287	0.99 (0.97–1.01)	1.02 (0.99–1.05)
Stressful life events	2.6 (3.54)	2.4 (2.65)	2.8 (2.81)	255	1.03 (0.03–1.14)	1.05 (0.90–1.23)
Response to unfair treatment	2.6 (0.76)	2.4 (0.63)	2.4 (0.49)	264	1.56^*^ **(1.01–2.42)**	0.82 (0.35–1.92)
Experience discrimination	0.7 (1.27)	0.7 (1.46)	0.8 (1.68)	260	0.97 (0.78–1.21)	1.02 (0.73–1.42)
Everyday discrimination	48.5 (10.04)	50.04 (7.39)	50.4 (6.29)	263	0.98 (0.95–1.01)	1.01 (0.94–1.08)
Interpersonal violence general	0.4 (0.65)	0.5 (0.73)	0.8 (0.75)	260	0.80 (0.50–1.26)	1.60 (0.88–2.92)
Education	3.1 (1.28)	3.4 (1.24)	3.4 (1.27)	268	0.84 (0.66–1.07)	0.97 (0.69–1.37)
	**Frequency (%)**					
Ethnicity/race				291		
Hispanic	15.2	69.7	15.2		0.97 (0.34–2.79)	2.39 (7.60–7.53)
Other	37.5	54.2	8.3		3.10^*^ **(1.20–8.0)**	1.69 (0.34–8.48)
Non-Hispanic Black	29.3	64	6.7		2.05^*^ **(1.07–3.95)**	1.15 (0.38–3.47)
White (Reference)	16	76.1	6.9			
Medicaid status				255		
Yes	21.6	71.2	7.2		1.18 (0.63–2.2)	0.89 (0.34–2.27)
No (Reference)	18.8	72.9	8.3			
Enrollment clinic				291		
Midwifery	14.9	79.3	5.7		0.46^*^ **(0.23–0.94)**	0.71 (0.34–1.47)
General OB	26.1	62.3	11.6		0.41 (0.14–1.18)	0.25 (0.06–1.15)
Community clinic (Reference)	21.2	75.8	3.0			
Currently employed				283		
Yes (Reference)	21.2	70.2	8.7			
No	22.7	70.7	6.7		1.06 (0.56–2.02)	0.77 (0.27–2.16)
Marital status				283		
Married/cohabitating	21.5	72.4	6.1		0.92 (0.52–1.62)	0.53 (0.22–1.26)
Single (Reference)	21.7	67.5	10.8			
Nulliparous				291		
Yes	18.8	69.8	11.5		0.82 (0.44–1.53)	1.90 (0.79–4.5)
No (Reference)	23.1	70.8	6.2			
Smoking during pregnancy (any)				283		
Yes	23.6	71.9	4.5		1.11 (0.6–2.03)	0.44 (0.15–1.36)
No (Reference)	20.6	69.6	9.8			
Receive WIC services				256		
Yes	21.8	66.7	11.5		1.3 (0.68–2.48)	2.19 (0.86–5.55)
No (Reference)	18.9	75.1	5.9			
Receive public assistance				256		
Yes	19.0	73.0	7.9		0.93 (0.45–1.93)	1.01 (0.35–2.92)
No (Reference)	20.2	72.0	7.8			
History of sexual assault				259		
Yes	16.7	66.7	16.7		0.86 (0.30–2.40)	3.5^*^ **(1.11–10.96)**
No (Reference)	21.4	73.4	5.2			
History of abuse				258		
Yes	15.0	76.3	8.8		0.59 (0.29–1.2)	1.45 (0.52–3.99)
No (Reference)	23.6	70.8	5.6			
Physical domestic violence				260		
Yes	23.1	69.2	7.7		1.18 (0.31–4.5)	1.25 (0.15–10.5)
No (Reference)	20.6	72.9	6.5			
Prenatal medical complications^a^				291		
Yes	17.6	67.6	14.7		0.56 (0.19–1.70)	2.31 (0.78–6.84)
No (Reference)	21.1	73.6	5.3			

a *Diagnosed with gestational hypertension, gestational diabetes, or pre-eclampsia*.

** *p < 0.01*,

* *p < 0.05*.

*The bold indicates that this value is statistically significant*.

Fewer factors differentiated women who received Adequate compared with Intensive care (Table 2). One notable finding was that an increased likelihood of Intensive care was associated with a history of sexual assault (*OR* = 3.50, 95% *CI* [1.12, 10.96], *p* = 0.031).

The multivariate model predicting Inadequate/Intermediate or Intensive care relative to Adequate care is presented in Table 3. The overall model was significant [*X*^2^_(20)_ = 36.32*, p* < 0.014]. Despite the number of significant bivariate associations in Table 2, the multivariate model indicated that, adjusting for all other factors in the model, only larger household size predicted less than adequate care (*OR* = 1.26, 95% *CI* [1.00, 1.59], *p* = 0.048). Table 2 also indicates that prenatal medical complications were significantly associated with Intensive care (*OR* = 4.93, 95% *CI* [1.28, 18.96], *p* = 0.02). The association between history of sexual assault and Intensive care was trivially changed from the bivariate to the adjusted multivariate model (OR of 3.5 and 3.38, with corresponding *p*-values of <0.05–0.056, respectively).

**Table 3:** Multinomial logistic regression analysis predicting inadequate and intensive prenatal care vs. adequate prenatal care (*n* = 240).

**Predictors**	**Less than adequate vs. adequate care**	**Intensive vs. adequate care**
	**OR (95% CI)**	**OR (95% CI)**
Age (years)	0.95 (0.87–1.03)	0.97 (0.86–1.09)
Household size (persons)	1.26 (1.00–1.59)^*^	0.88 (0.55–1.40)
Pregnancy anxiety (regarding baby)	0.97 (0.93–1.00)	1.01 (0.96–1.06)
Social support	0.99 (0.97–1.01)	1.00 (0.97–1.05)
Response to unfair treatment	1.21 (0.72–2.04)	0.90 (0.34–2.37)
Ethnicity/race		
Hispanic	0.51 (0.13–2.06)	2.86 (0.62–13.08)
Other	5.63 (1.64–19.37)^**^	3.84 (0.56–26.25)
Non-Hispanic Black	0.72 (0.29–1.77)	0.85 (0.19–3.76)
White (Reference)		
History of sexual assault		
Yes	0.33 (0.08–1.39)	3.38 (0.97–11.75)
No (Reference)		
Prenatal medical complications		
Yes	1.06 (0.32–3.47)	4.93 (1.28–18.96)^*^
No (Reference)		

** *p < 0.01*,

* *p < 0.05*.

*The bold indicates that this value is statistically significant*.

### Postpartum Clinic Attendance

Of the 291 participants with prenatal attendance data, 277 also had postnatal attendance data, of whom 89.5% completed at least one postnatal visit within 12 weeks after childbirth. Table 4 identifies many factors associated with completion of a postpartum visit. Postpartum visit status was not significantly associated with either perinatal outcome that was associated with prenatal care utilization, that is, gestational age or birth weight (*p*'s > 0.1).

**Table 4:** Bivariate logistic regression analysis predicting completion of postpartum visit.

**Predictors**	**Completed visit**	**Did not complete visit**		**Did vs. did not complete visit**
	**frequency (%)**		***N***	**(df) X^**2**^**
Age (years)	29.04 (4.57)	27.48 (4.84)	277	(1, 275) 2.98
Pre-pregnancy BMI (kg/m^2^)	28.20 (7.17)	28.73 (6.52)	277	(1, 275) 0.14
Poverty income ratio	3.88 (4.15)	2.30 (2.01)	231	(1, 229) 3.50
Household size (persons)	2.14 (1.33)	3.10 (2.18)	271	(1, 269) 11.62^**^
Employed hours/week	35.50 (12.03)	33.47 (17.09)	197	(1, 195) 0.41
Depressive symptoms	6.03 (4.54)	5.15 (4.46)	275	(1, 273) 0.93
Pregnancy anxiety (regarding baby)	21.70 (11.11)	15.66 (5.51)	277	(1, 275) 8.32^**^
Pregnancy anxiety (regarding labor)	19.98 (9.61)	17.79 (9.20)	277	(1, 275) 1.36
Social support	101.03 (15.74)	88.83 (19.53)	257	(1, 255) 12.46^**^
Worry symptoms	45.10 (12.70)	39.66 (11.80)	275	(1, 253) 4.53^*^
Stressful life events	2.52 (2.95)	2.04 (1.99)	250	(1, 248) 0.63
Response to unfair treatment	2.47 (0.65)	2.44 (0.71)	259	(1, 257) 0.05
Experience discrimination	0.75 (1.46)	0.48 (1.26)	255	(1, 253) 0.78
Everyday discrimination	49.52 (8.16)	50.92 (6.24)	258	(1, 256) 0.69
Interpersonal violence general	0.48 (0.73)	0.46 (0.59)	255	(1, 253) 0.02
Education	3.4 (1.22)	2.92 (1.41)	255	(1, 253) 3.30
	**Frequency (%)**			**(df)** ***X***^**2**^
Ethnicity/race			277	(3) 6.53
Hispanic	93.3	6.7		.
Other	83.3	16.7		
Non-Hispanic Black	82.9	17.1		
White	92.8	7.2		
Medicaid status			248	(1) 4.73^*^
No	93.6	6.4		
Yes	85.2	14.8		
Enrollment clinic			277	(2) 1.15
Midwifery	90.6	9.4		
Community clinic	87.5	12.5		
General OB	92.2	7.8		
Currently employed			270	(1) 4.28^*^
Yes	91.5	8.5		
No	82.6	17.4		
Marital status			270	(1) 2.51
Married/cohabiting	91.8	8.2		
Single	85.7	14.3		
Nulliparous			277	(1) 3.73
Yes	94.6	5.4		
No	87.0	13.0		
Smoking during pregnancy (any)			270	(1) 0.55
No	90.2	9.8		
Yes	87.2	12.8		
Receive WIC services			249	(1) 0.37
No	90.8	9.2		
Yes	88.4	11.6		
Receive public assistance			249	(1) 0.18
No	90.4	9.6		
Yes	88.5	11.5		
History of sexual assault			254	(1) 0.31
Yes	93.3	6.7		
No	90.2	9.8		
History of abuse			253	(1) 0.49
No	91.4	8.6		
Yes	88.6	11.4		
Physical domestic violence			255	(1) 1.42
Yes	90.1	9.9		
No	100.0	0.0		
Childhood sexual abuse			256	(1) 0.26
No	91.0	9.0		
Yes	88.2	11.8		
Prenatal medical complications			277	(1) 3.93
No	88.3	11.7		
Yes	100.0	0.0		

** *p < 0.01*,

* *p < 0.05*.

*The bold indicates that this value is statistically significant*.

Likelihood of completing a postnatal visit was significantly associated with smaller household membership, elevated pregnancy-related anxiety, elevated general anxiety/worry, increased social support, being employed, and Medicaid status. Postpartum visit completion was also significantly associated with prenatal care utilization: 73.3% of those with Inadequate prenatal care completed a postpartum visit compared with 93.4% of those in the Adequate and 100% of those in the Intensive KI group [*X*^2^_(2)_ = 22.28, *p* < 0.001].

Results from the multivariate model (Table 5) indicated that prenatal care utilization independently predicted completion of a postpartum visit, as did elevated social support and employment status; none of the other variables significantly predicted postpartum visit independent of other variables in the model.

**Table 5:** Multivariate binary logistic regression analysis predicting completion of postpartum visit.

**Predictors**	**Did vs. did not complete postpartum visit**
	**OR (95% CI)**
Household size	0.90 (0.68–1.17)
Pregnancy anxiety (regarding baby)	1.05 (0.98–1.12)
Social support	1.04^**^ **(1.01–1.07)**
Worry symptoms	1.04 (1–1.09)
KI category	
Inadequate	0.21^**^ **(0.08–0.57)**
Adequate/intensive (Reference)	
Medicaid status	
Yes	0.90 (0.30–2.73)
No (Reference)	
Currently employed	
Yes	2.86^*^ **(1.02–8.03)**
No (Reference)	

** *p < 0.01*,

* *p < 0.05*.

*The bold indicates that this value is statistically significant*.

## Discussion

The current study leveraged several design strengths of a large, prospective, longitudinal pregnancy cohort study to test core components of a biopsychosocial model of perinatal health care utilization. Congruent with predictions from a biopsychosocial model, perinatal health care utilization was associated with a diversity of social context, psychological, and socio-demographic factors. Multivariate model results highlighted the overlapping nature of these predictors, and identified social support, family context (size), anxiety, and trauma as among the more robust predictors of perinatal care utilization. We consider how the findings advance research in the area, and then consider several possible clinical applications for increasing perinatal care utilization.

The portion of women who received adequate or intensive care in our study, 78%, is comparable to other studies, including those with diverse samples (10). Our reported rate of inadequate care (as distinct from intermediate care) of 2% matches other studies that have been actively following women in pregnancy, such as the rate of 2.4% in one controlled trial in pregnancy (26). On the other hand, this rate is lower than other studies not requiring participant engagement in an intensive prenatal assessment, e.g., rates of 10% or higher have been reported (7, 19). As regards postnatal care utilization, comparatively few of our study participants, ~10%, did not complete at least one visit within the recommended timeframe. Rates twice as high have been reported (28). The explanation may also reflect a possible bias of engagement in a research study. Participation in the research study did provide incentives (compensation was provided for prenatal research visits that occurred in the same clinic women received their prenatal care) and perhaps an implicit social support or other inducement to attend perinatal health care visits. Alternatively, study participation may not have had any causal role in increasing perinatal care utilization, e.g., to the extent that women who are already very engaged in their prenatal care may be more disposed to participate in a research study on pregnancy and child health. We were unable to test this hypothesis directly because we were not able to compare perinatal care utilization in those women who refused participation in the study.

A starting point for our analyses predicting prenatal care utilization is the observation that there were significant group differences in both gestational age and birth weight among the Adequate, Inadequate/Intermediate, and Intensive care utilization groups. Those who received adequate care according to ACOG guidelines had the most positive perinatal outcomes. The magnitude of effect was not large but nonetheless notable in this healthy sample of women, all of whom started prenatal care by the first trimester. The findings underscore the value in targeting prenatal care utilization for improving health outcomes even in normal to low-risk samples. Postnatal care visit utilization was not associated with these same perinatal outcomes but may be more relevant for maternal postnatal health and screening, which was not assessed in this study.

The biopsychosocial model proposes that health and health care is best seen as a system embedded in, and reflective of, the complex social context. We operationalized and tested this model by analyzing potential sources of variability in perinatal health care utilization. The findings provide broad-based support for the model: we identified a wide range of social and personal psychological factors that, at least in bivariate analyses, associated with pre- and post-natal care utilization. These factors included affective symptoms of anxiety and worry, which generally increased care utilization; social support, which promoted adequate pre- and post-natal care; and family size, which decreased likelihood of adequate perinatal care and may index something of the practical difficulties of obtaining care or the increased organization and arrangements needed to access care. In fact, social and personal factors were more reliable predictors of pre- and post-natal health care than conventional health markers such as BMI or other health behaviors; the positive association between prenatal complications and intensive prenatal care was the exception to this general pattern. Social and psychological factors were also more reliably associated with perinatal care utilization than socio-economic factors, including education, income, and social services use. These findings emphasize that health care access or utilization is a product of social and behavioral forces. The predictability of postnatal care utilization from prenatal care utilization may reflect stable personal traits or social context.

Several additional findings deserve particular attention. As reported by many others, women who identify as non-Hispanic Black were more likely to experience less than adequate prenatal care in bivariate analyses; this did not extend to postpartum care, however. This association for prenatal care was not confirmed in multivariate analyses that considered multiple and confounding social and socio-demographic factors. This suggests that the other variables in the model accounted for, or were at least confounded with, race/ethnic differences in care utilization. The implication is that the widely-reported differential take-up of or access to health care in certain minority groups may be explicable in terms of confounding social and demographic factors that may be plausible targets for improving perinatal health care utilization. That, too, is consistent with the biopsychosocial model's emphasis on the embedded and confounded natures of risks.

A second findings concerns the role of trauma history. Individuals who reported sexual trauma in the course of the research study were more likely to experience intensive prenatal care. This was contrary to previous findings associating past and current IPV with late or inadequate prenatal care (24). The implication is that sexual trauma history is associated with increased care seeking rather than providers responding differentially to women with a trauma history (although we are unable to rule out the latter possibility). In this context, it is important to note that intensive care is not associated with better outcomes, at least as regards birth weight and gestational age.

The third finding demonstrated that, in the bivariate analysis, pregnancy-related anxiety specific to concerns about the baby were related to a *decreased* likelihood of less than adequate prenatal care, as well as a *higher* likelihood of completing a postpartum visit. Ours is the first study we are aware of that examined pregnancy-specific anxiety in relation to prenatal care utilization patterns across pregnancy and beyond. Pregnant women who have anxiety related to the well-being of their child may be more inclined to desire assistance and assurance from professional care providers, thereby making them less likely to deviate from their attendance from a recommended schedule of prenatal care visits. This pattern of anxiety and care utilization is well-established outside of the perinatal care context (40), and may have implications for improving clinical outcomes while also reducing health care costs.

Several limitations of the study should be noted. The first is that the findings may not generalize to all samples of interest. We chose to study a generally health group of women who initiated prenatal care by the first trimester to examine biopsychosocial influences unconfounded with medical risk. Our focus on the current cohort also meant that the women were followed more closely (as a function of study participation, and they did receive compensation for prenatal research visits) than would be typical. In addition, although the study was comparatively large, it was not positioned to examine perinatal care utilization for all ethnic/minority groups; in particular, our finding of increased likelihood of less than adequate care among the small and diverse participants who did not identify as Hispanic, non-Hispanic Black or non-Hispanic white requires further investigation. Third, we did not have sufficient data to examine clinic characteristics that may explain variation in perinatal care utilization; our explanatory focus was limited to characteristics of the women in the study. Finally, the Kotelchuck Index, although well-validated and commonly used in research, is limited in its ability to measure care quality because it does not capture the actual visit content. Elements of the visit that are crucial for determining care quality—provider-patient communication, patient satisfaction, and health outcomes—are not considered in this index. It is also important to consider the possibility that patients who have experienced racial discrimination in maternal health care settings may have been reluctant to reveal their experiences in a survey administered in the context of such settings. Research incorporating mixed-methods approaches that include qualitative interviews may be valuable in providing additional insight into the nature of extent of care quality. Set against these limitations were several strengths of the study, including a detailed assessment of social context, psychological, and demographic factors; a multivariate approach that considered overlapping and competing predictors of less than adequate and intensive care; and a consideration of care utilization in pregnancy and the early postpartum period.

Several clinical applications are suggested by the findings. For example, the results concerning race and response to unfair treatment suggest that women who prefer to avoid confronting structural racism are more vulnerable to inadequate prenatal care, which may make the hospital environment uncomfortable. The implication may be that outreach and trust-building experiences may be needed to improve health care utilization. Additionally, increased psychosocial screening at prenatal appointments—particularly in the early stages of pregnancy—may identify patients at elevated risk for poor attendance or worse birth outcomes, and could be clinically and cost-effective. Targeting interpersonal violence, which is now routine in most settings, as well as affective symptoms and family setting may also identity those at greatest risk for inadequate care. Third, in contrast to socio-demographic or socio-economic indicators, many of the predictors of perinatal care utilization identified here, such as affective symptoms, are modifiable, and may be responsive to brief targeted interventions to complement routine obstetric care.

## Data Availability Statement

The raw data supporting the conclusions of this article will be made available by the authors, without undue reservation.

## Ethics Statement

The studies involving human participants were reviewed and approved by University of Rochester Medical Center Research Subjects Review Board. The patients/participants provided their written informed consent to participate in this study.

## Author Contributions

ZD, TO'C, and LP were involved in the literature review and manuscript writing. JB, CI, and ZD did the medical record abstraction and data cleaning. SB, ZD, and TO'C conducted data analyses. JB, JM, and MB formatted tables and figures. EPr, EPo, LT, KB, EB, and RM provided guidance from a clinical perspective and they also provided comments on multiple rounds of drafts. All authors contributed to the article and approved the submitted version.

## Funding

Funding was provided by NIH Grants UG/UH OD023349, HD083369, and the Wynne Center for Family Research. Additional support was provided by TL1TR000096.

## Conflict of Interest

The authors declare that the research was conducted in the absence of any commercial or financial relationships that could be construed as a potential conflict of interest.

## Publisher's Note

All claims expressed in this article are solely those of the authors and do not necessarily represent those of their affiliated organizations, or those of the publisher, the editors and the reviewers. Any product that may be evaluated in this article, or claim that may be made by its manufacturer, is not guaranteed or endorsed by the publisher.

## References

[1] Engel GL. The need for a new medical model: a challenge for biomedicine. Science. (1977) 196:129–36. 10.1126/science.847460 847460

[2] Ghaemi SN. The rise and fall of the biopsychosocial model. Br J Psychiatry. (2009) 195:3–4. 10.1192/bjp.bp.109.063859 19567886

[3] MacDorman MF Declercq E Cabral H Morton C. Is the United States maternal mortality rate increasing? Disentangling trends from measurement issues short title: US maternal mortality trends. Obstetrics Gynecol. (2016) 128:447. 10.1097/AOG.0000000000001556 27500333 PMC5001799

[4] World Health Organization. Strategies Towards Ending Preventable Maternal Mortality (EPMM). (2015). Available online at: https://apps.who.int/iris/bitstream/handle/10665/153544/9789241508483_eng.pdf

[5] O'Connor TG Heron J Golding J Beveridge M Glover V. Maternal antenatal anxiety and children's behavioural/emotional problems at 4 years. Report from the Avon Longitudinal Study of Parents and Children. Br J Psychiatry. (2002) 180:502–8. 10.1192/bjp.180.6.502 12042228

[6] Kotelchuck M. The adequacy of prenatal care utilization index: its US distribution and association with low birthweight. Am J Public Health. (1994) 84:1486–9. 10.2105/AJPH.84.9.1486 8092377 PMC1615176

[7] Partridge S Balayla J Holcroft CA Abenhaim HA. Inadequate prenatal care utilization and risks of infant mortality and poor birth outcome: a retrospective analysis of 28,729,765 U.S. deliveries over 8 years. Am J Perinatol. (2012) 29:787–73. 10.1055/s-0032-1316439 22836820

[8] Gadson A Akpovi E Mehta PK. Exploring the social determinants of racial/ethnic disparities in prenatal care utilization and maternal outcome. Semin Perinatol. (2017) 41:308–17. 10.1053/j.semperi.2017.04.008 28625554

[9] Appel HB Nguyen PD. Eliminating racial and ethnic disparities in behavioral health care in the U.S. J Health Soc Sci. (2020) 5:441–8. 10.19204/2020/lmnt7

[10] Cox R Zhang L Zotti M Graham J. Prenatal care utilization in mississippi: racial disparities and implications for unfavorable birth outcomes. Matern Child Health J. (2011) 15:931–42. 10.1007/s10995-009-0542-6 19943096

[11] Fryer K Munoz MC Rahangdale L Stuebe AM. Multiparous Black and Latinx women face more barriers to prenatal care than white women. J Racial Ethnic Health Disparities. 8:80–7 (2021). 10.1007/s40615-020-00759-x 32333378

[12] Slaughter-Acey JC Caldwell CH Misra DP. The influence of personal and group racism on entry into prenatal care among-African American Women. Women's Health Issues. (2013) 23:e381–7. 10.1016/j.whi.2013.08.001 24041828 PMC3845454

[13] Murali V Oyebode F. Poverty, social inequality and mental health. Adv Psychiatric Treatment. (2004) 10:216–24. 10.1192/apt.10.3.216

[14] Nadal KL Erazo T King R. Challenging definitions of psychological trauma: Connecting racial microaggressions and traumatic stress. J Soc Action Counsel Psychol. (2019) 11:2–16. 10.33043/JSACP.11.2.2-16

[15] Kelly RH Danielsen BH Golding JM Anders TF Gilbert WM Zatzick DF. Adequacy of prenatal care among women with psychiatric diagnoses giving birth in California in 1994 and 1995. Psychiatric Services. (1999) 50:1584–90. 10.1176/ps.50.12.1584 10577877

[16] Kim HG Mandell M Crandall C Kuskowski MA Dieperink B Buchberger RL. Antenatal psychiatric illness and adequacy of prenatal care in an ethnically diverse inner-city obstetric population. Arch Women's Mental Health. (2006) 9:103–7. 10.1007/s00737-005-0117-5 16380813

[17] Sidebottom AC Hellerstedt WL Harrison PA Jones-Webb RJ. Prenatal care: associations with prenatal depressive symptoms and social support in low-income urban women. Arch Women's Mental Health. (2017) 20:633–44. 10.1007/s00737-017-0730-0 28578453

[18] Pagnini DL Reichman NE. Psychosocial factors and the timing of prenatal care among women in New Jersey's HealthStart program. Family Planning Perspect. (2000) 32:56–64. 10.2307/2648213 10779236

[19] Heaman MI Martens PJ Brownell MD Chartier MJ Derksen SA Helewa ME. The association of inadequate and intensive prenatal care with maternal, fetal, and infant outcomes: a population-based study in Manitoba, Canada. J Obstetrics Gynaecol Canada. (2019) 41:947–59. 10.1016/j.jogc.2018.09.006 30639165

[20] Johnson RL Roter D Powe NR Cooper LA. Patient race/ethnicity and quality of patient-physician communication during medical visits. Am J Public Health. (2004) 94:2084–90. 10.2105/AJPH.94.12.2084 15569958 PMC1448596

[21] Wolf ER Donahue E Sabo RT Nelson BB Krist AH. Barriers to attendance of prenatal and well-child visits. Academic Pediatrics. (2021) 21:955–60. 10.1016/j.acap.2020.11.025 33279734 PMC8172669

[22] Cha S Masho SW. Intimate partner violence and utilization of prenatal care in the United States. J Interpers Violence. (2014) 29:911–27. 10.1177/0886260513505711 24203982

[23] Musa A Chojenta C Geleto A Loxton D. The associations between intimate partner violence and maternal health care service utilization: a systematic review and meta-analysis. BMC Women's Health. (2019) 19:1–14. 10.1186/s12905-019-0735-0 30808353 PMC6390526

[24] Jamieson B. Exposure to interpersonal violence during pregnancy and its association with women's prenatal care utilization: a meta-analytic review. Trauma Violence Abuse. (2020) 21:904–21. 10.1177/1524838018806511 30322355

[25] Sebert Kuhlmann AK Foggia J Fu Q Sierra M. Intimate partner violence as a predictor of antenatal care service utilization in Honduras. Pan Am J Public Health. (2017) 41:e104. 10.26633/RPSP.2017.104 28902264 PMC6660898

[26] Magriples U Kershaw TS Rising SS Massey Z Ickovics JR. Prenatal health care beyond the obstetrics service: Utilization and predictors of unscheduled care. Am J Obstetrics Gynecol. (2008) 198:75.e1–7. 10.1016/j.ajog.2007.05.040 18166312 PMC2276882

[27] American College of Obstetricians and Gynecologists. Optimizing Postpartum Care: Committee Decision (Number 736). (2018). Available online at: https://www.acog.org/clinical/clinical-guidance/committee-opinion/articles/2018/05/optimizing-postpartum-care

[28] Baldwin MK Hart KD Rodriguez MI. Predictors for follow-up among postpartum patients enrolled in a clinical trial. Contraception. (2018) 98:228–31. 10.1016/j.contraception.2018.04.016 29750924 PMC6129217

[29] Bengiamin MI Capitman JA Ruwe MB. Disparities in initiation and adherence to prenatal care: impact of insurance, race-ethnicity and nativity. Matern Child Health J. (2010) 14:618–24. 10.1007/s10995-009-0485-y 19557508

[30] Blackmore ER Gustafsson H Gilchrist M Wyman C O'Connor TG. Pregnancy-related anxiety: evidence of distinct clinical significance from a prospective longitudinal study. J Affect Disord. (2016)197:251–8. 10.1016/j.jad.2016.03.008 26999549 PMC4837058

[31] Services UD of HH. Prior HHS Poverty Guidelines and Federal Register References. (2021). Available online at: https://aspe.hhs.gov/prior-hhs-poverty-guidelines-and-federal-register-references

[32] Cohen S Wills TA. Stress, social support, and the buffering hypothesis. Psychol. Bull. (1985) 98:310–57. 10.1037/0033-2909.98.2.310 3901065

[33] Meyer TJ Miller ML Metzger RL Borkovec TD. Development and validation of the Penn state worry questionnaire. Behav Res Ther. (1990) 28:487–95. 10.1016/0005-7967(90)90135-6 2076086

[34] Cox JL Holden JM Sagovsky R. Detection of postnatal depression: development of the 10-item Edinburgh postnatal depression scale. Br J Psychiatry. (1987) 150:782–6. 10.1192/bjp.150.6.782 3651732

[35] McFarlane J Parker B Soeken K Bullock L. Assessing for abuse during pregnancy: severity and frequency of injuries and associated entry into prenatal care. JAMA. (1992) 267:3176–8. 10.1001/jama.1992.03480230068030 1593739

[36] Robertson Blackmore E Mittal M Cai X Moynihan JA Matthieu MM O'Connor TG. Lifetime exposure to intimate partner violence and proinflammatory cytokine levels across the perinatal period. J Women's Health. (2016) 25:1004–13. 10.1089/jwh.2015.5261 26744816 PMC5069724

[37] Barnett BEW Hanna B Parker G. Life event scales for obstetric groups. J Psychosomatic Res. (1983) 27:313–20. 10.1016/0022-3999(83)90054-5 6620208

[38] Krieger N Smith K Naishadham D Hartman C Barbeau EM. Experiences of discrimination: validity and reliability of a self-report measure for population health research on racism and health. Soc Sci Med. (2005) 61:1576–96. 10.1016/j.socscimed.2005.03.006 16005789

[39] IBM. IBM SPSS - IBM Analytics. Armonk, NY: IBM SPSS Software (2016).

[40] Horenstein A Heimberg RG. Anxiety disorders and healthcare utilization: a systematic review. Clin Psychol Rev. (2020) 81:101894. 10.1016/j.cpr.2020.101894 32818687

