# Supplementary material for: The Biopsychosocial Model and Perinatal Health Care: Determinants of Perinatal Care in a Community Sample
Source: Front Psychiatry. 2021 Nov 17;12:746803. doi: 10.3389/fpsyt.2021.746803 (PMC8635705; doi:10.3389/fpsyt.2021.746803)
Supplement: Supplementary file 1 [file Table_1.docx]

**Supplementary Table 1. Bivariate Logistic regression analysis predicting low and high rate of no-shows vs. zero no-shows.**

As noted in the text, the outcome of each scheduled routine clinic visits was categorized in the medical record as completed, cancelled/rescheduled, or no show. We include analyses of no-show rates because they may be of interest and are a contrast to the KI. The table displays results of bivariate analyses using logistic regressions between biopsychosocial predictors and three levels of prenatal no-show rates. “Zero” indicates no no-show results from any scheduled routine visit. The low rate was defined by a ratio of no-shows to completed prenatal visits ranging from .01 through .19; the high rate was defined by a ratio of no-shows to completed prenatal visits greater than .2. This distinction was determined by dividing the group with any no shows roughly at the median to create similar sizes for each group. The average number of no-shows was .57 (SD = 1.37) and the no-show rate was significantly correlated with the Kotelchuck Index (*r* = -.433, p<.001).

The predictors were the same as those used in the Kotelchuck Index (KI) and postpartum visit analyses. As demonstrated by the means and frequencies in the table, the levels of most predictors have a linear relationship with no-show rate. Findings from the bivariate analyses indicate that social support, household size, enrollment clinic and pregnancy specific anxiety regarding baby were significant predictors of no-show rate. Variables that uniquely predicted no-show rate (and not prenatal or postpartum clinic attendance) were also identified: poverty-income ratio, depressive symptoms, stressful life events, experiences of discrimination, education, marital status, smoking, and receiving WIC or public assistance. No-show rate was also significantly associated with birth weight and gestational age.

| **Predictors** | **Zero No-shows** | **Low No-show rate** | **High No-show rate** |  | **Low No-show rate vs. Zero No-shows** | **High No-show rate vs. Zero No-shows** |
| --- | --- | --- | --- | --- | --- | --- |
|  | **Mean (SD)** | | | **N** | **OR (95% CI)** | **OR (95% CI)** |
| Age (years) | 29.7 (4.16) | 28.6 (4.75) | 23.6 (4.34) | 290 | .94 (.87-1.01) | **.71** (.63-.8)** |
| Pre-pregnancy BMI (kg/m^2^) | 27.8 (6.98) | 28.8 (6.56) | 30.17 (8.44) | 290 | 1.02 (.98-1.07) | 1.04 (.99-1.10) |
| Poverty income ratio | 4.1 (4.28) | 3.3 (2.24) | 1.2 (.58) | 242 | .92 (.80-1.05) | **.28** (.15-.54)** |
| Household size (persons) | 2.1 (1.29) | 2.2 (1.28) | 3.2 (2.37) | 283 | 1.06 (.83-1.34) | **1.44** (1.16-1.8)** |
| Employed hours/week | 35.3 (12.68) | 38.3 (11.45) | 31.5 (11.84) | 208 | 1.02 (.99-1.05) | .97 (.93-1.02) |
| Depressive symptoms | 5.5 (4.28) | 7.2 (4.79) | 7.0 (5.20) | 288 | **1.08* (1.01-1.16)** | 1.07 (.99-1.17) |
| Pregnancy anxiety (regarding baby) | 22.5 (11.12) | 19.3 (10.01) | 15.8 (10.05) | 289 | .97 (.94-.1.00) | **.92** (.87-.97)** |
| Pregnancy anxiety (regarding labor) | 20.5 (9.39) | 17.8 (9.25) | 18.0 (10.80) | 289 | .97 (.93-1.00) | .97 (.93-1.02) |
| Social support | 101.6 (15.7) | 96.8 (17.92) | 89.9 (18.18) | 269 | .98 (.96-1.0) | **.97** (.94-.99)** |
| Worry symptoms | 44.7 (13.11) | 44.7 (11.31) | 43.4 (10.96) | 288 | 1.00 (.98-1.03) | .99 (.96-1.02) |
| Stressful life events | 2.2 (2.47) | 3.0 (3.20) | 4.1 (4.28) | 262 | 1.11 (.99-1.25) | **1.22** (1.07-1.39)** |
| Response to unfair treatment | 2.4 (.62) | 2.5 (.63) | 2.9 (.83) | 271 | 1.15 (.69-1.93) | **2.44** (1.34-4.44)** |
| Experience discrimination | .6 (1.26) | 1.0 (1.68) | 1.5 (1.90) | 267 | 1.21 (.97-1.51) | **1.41** (1.12-1.79)** |
| Everyday discrimination | 50.4 (7.34) | 48.2 (9.50) | 47.3 (8.51) | 270 | .97 (.93-1.00) | .96 (.92-1.00) |
| Interpersonal violence general | .4 (.70) | .6 (.75) | .6 (.78) | 267 | 1.24 (.79-1.96) | 1.37 (.79-2.4) |
| Education | 3.6 (1.19) | 3.0 (1.24) | 2.0 (.69) | 267 | **.67** (.50-.89)** | **.21** (.10-1.42)** |
|  | **(%)** | | |  |  |  |
| Ethnicity/ Race |  |  |  | 290 |  |  |
| Hispanic | 66.7 | 20.0 | 13.3 |  | 2.74 (.96-7.81) | **7.3** (1.69-31.51)** |
| Other | 75.0 | 20.8 | 4.2 |  | 2.54 (.83-7.75) | 2.03 (.22-19.15) |
| Non-Hispanic Black | 45.7 | 27.1 | 27.1 |  | **5.42** (2.52-11.67)** | **21.67** (6.9-68.04)** |
| White (Reference) | 88.0 | 9.6 | 2.4 |  |  |  |
| Medicaid status |  |  |  | 258 |  |  |
| Yes | 64.5 | 17.3 | 18.2 |  | 1.69 (.84-3.37) | **17.75** (4.03-78.14)** |
| No (Reference) | 85.1 | 13.5 | 1.4 |  |  |  |
| Enrollment Clinic |  |  |  | 290 |  |  |
| Midwifery | 85.2 | 13.6 | 1.1 |  | .51 (.24-1.08) | **.04** (.01-.32)** |
| General OB | 86.6 | 11.9 | 1.5 |  | .44 (.19-1.04) | **.06** (.01-.42)** |
| Community Clinic (Reference) | 61.5 | 19.3 | 19.3 |  |  |  |
| Currently employed |  |  |  | 282 |  |  |
| Yes (Reference) | 76.9 | 16.5 | 6.6 |  |  |  |
| No | 68.6 | 12.9 | 18.6 |  | .87 (.39-1.94) | **3.15** (1.39-7.16)** |
| Marital Status |  |  |  | 283 |  |  |
| Married/cohabitating | 88.1 | 10.1 | 1.8 |  | **.28** (.14-.54)** | **.06** (.02-.2)** |
| Single (Reference) | 56.5 | 23.5 | 20.0 |  |  |  |
| Nulliparous |  |  |  | 289 |  |  |
| Yes | 78.8 | 14.1 | 7.1 |  | .77 (.39-1.53) | .59 (.24-1.44) |
| No (Reference) | 72.1 | 16.8 | 11.1 |  |  |  |
| Smoking during pregnancy (any) |  |  |  | 282 |  |  |
| Yes | 67.4 | 16.9 | 15.7 |  | 1.30 (.65-2.60) | **2.71* (1.20-6.11)** |
| No (Reference) | 78.2 | 15.0 | 6.7 |  |  |  |
| Receive WIC services |  |  |  | 259 |  |  |
| Yes | 70.5 | 12.5 | 17.0 |  | .86 (.40-1.84) | **4.70** (1.83-12.11)** |
| No (Reference) | 79.5 | 16.4 | 4.1 |  |  |  |
| Receive public assistance |  |  |  | 259 |  |  |
| Yes | 65.6 | 11.5 | 23.0 |  | .86 (.36-2.10) | **6.91** (2.71-17.61)** |
| No (Reference) | 79.8 | 16.2 | 4.0 |  |  |  |
| History of sexual assault |  |  |  | 266 |  |  |
| Yes | 70.0 | 16.7 | 13.3 |  | 1.24 (.44-3.50) | 1.83 (.57-5.87) |
| No (Reference) | 77.1 | 14.8 | 8.1 |  |  |  |
| History of abuse |  |  |  | 265 |  |  |
| Yes | 72.0 | 17.1 | 11.0 |  | 1.37 (.67-2.81) | 1.57 (.64-3.82) |
| No (Reference) | 78.7 | 13.7 | 7.7 |  |  |  |
| Physical domestic violence |  |  |  | 267 |  |  |
| Yes | 69.2 | 23.1 | 7.7 |  | 1.76 (.45-6.80) | .99 (.12-8.14) |
| No (Reference) | 76.8 | 14.6 | 8.7 |  |  |  |
| Prenatal Medical Complications |  |  |  | 290 |  |  |
| Yes | 72.7 | 21.2 | 6.1 |  | 1.44 (.58-3.57) | .62 (.14-2.76) |
| No (Reference) | 74.7 | 15.2 | 10.1 |  |  |  |
| **Birth Outcomes** | **Zero No-shows** | **Low No-show rate** | **High No-show rate** |  | **Low No-show rate vs. Zero No-shows** | **High No-show rate vs. Zero No-shows** |
|  | **Mean (SD)** | | | **N** | **OR (95% CI)** | **OR (95% CI)** |
| Gestational Age (wks) | 39.7 (1.36) | 38.9 (1.86) | 38.9 (1.47) | 216 | **.73** (.60-.90)** | **.73* (.57-.93)** |
| Birth Weight (g) | 3437.2 (503.99) | 3156.9 (613.40) | 3095.4 (373.98) | 216 | **.99** (.99-1.0)** | **.99** (.99-1.0)** |

Note. N’s for the groups are 216 for Zero, 46 for Low no-show rate, and 28 for high no-show rate. ** p<.01, * p<.05.
